# HOW DO FORMER DEMENTIA CAREGIVERS STRUCTURE HIGH- AND LOW-POINT NARRATIVES?

**DOI:** 10.1093/geroni/igad104.1140

**Published:** 2023-12-21

**Authors:** Tara Matta-Singh, Shubam Sharma, Talha Ali, Amanda Piechota, Anissa Abboud, Terri Fried, Joan Monin, Emily Mroz

**Affiliations:** University of South Florida, Tampa, Florida, United States; Kennesaw State University, Kennesaw, Georgia, United States; Tufts University, Medford, Massachusetts, United States; Yale School of Public Health, New Haven, Connecticut, United States; Yale School of Public Health, New Haven, Connecticut, United States; Yale School of Medicine, New Haven, Connecticut, United States; Yale School of Public Health, New Haven, Connecticut, United States; Yale School of Medicine, New Haven, Connecticut, United States

## Abstract

Former caregivers of people living with dementia often develop and structure narratives of their lived experiences as caregivers in terms of subjective high and low points. We utilized thematic analysis to examine narratives of these high and low points in interviews with 32 former caregivers of a parent living with dementia. Across both high and low points, caregivers often structured their narratives using three types of “elaboration,” that is, points not necessary to recall a factual event, but which help convey deeper meaning. Caregivers who utilized comparisons contrasted their caregiving experiences with other individuals or caregiving situations to help make sense of their own experience. When participants made connections to earlier life, they linked caregiving to prior experiences such as growing up with their parents. Shifting the focal point occurred where caregivers moved the narrative towards or away from a certain positive or negative storyline, unprompted. We discuss how caregivers who utilize elaborations adaptively (e.g., to tell a fuller story that reinforces how their caregiving relationship is beneficial) may come to better make sense of their experience which can alleviate some distress. We also discuss how elaborations that emphasize incompetency or promote focus on difficult moments may threaten the caregivers’ sense of well-being, leading to psychological harm. We conclude by introducing concrete narrative strategies that can help former caregivers use elaborations in adaptive ways, such as comparing difficult caregiving experiences to other difficulties that they have overcome to re-write narratives surrounding personal incompetency in caregiving.

